# Psychiatric Comorbidities among People Who Inject Drugs in Hai Phong, Vietnam: The Need for Screening and Innovative Interventions

**DOI:** 10.1155/2018/8346195

**Published:** 2018-10-04

**Authors:** Khue Pham Minh, Roselyne Vallo, Huong Duong Thi, Oanh Khuat Thi Hai, Don C. Des Jarlais, Marianne Peries, Sao Mai Le, Delphine Rapoud, Catherine Quillet, Tuyet Thanh Nham Thi, Giang Hoang Thi, Jonathan Feelemyer, Vinh Vu Hai, Jean-Pierre Moles, Xanh Pham Thu, Didier Laureillard, Nicolas Nagot, Laurent Michel, DRIVE Study Team

**Affiliations:** ^1^Hai Phong University of Medicine and Pharmacy, Hai Phong, Vietnam; ^2^Pathogenesis and Control of Chronic Infections, Inserm, University of Montpellier, Etablissement Français du Sang, Montpellier, France; ^3^Supporting Community Development Initiatives, Hanoi, Vietnam; ^4^Icahn School of Medicine at Mount Sinai, New York City, USA; ^5^Infectious Diseases Department, Viet Tiep Hospital, Hai Phong, Vietnam; ^6^Hai Phong Department of Health, Hai Phong, Vietnam; ^7^Infectious Diseases Department, Caremeau University Hospital, Nîmes, France; ^8^CESP/Inserm 1018, Pierre Nicole Center, French Red Cross, Paris, France

## Abstract

The objective of this study is to describe psychiatric comorbidities, associated factors, and access to psychiatric assessment and care in a cohort of people who inject drugs (PWID) in Hai Phong, Vietnam. Mental health was assessed after 12 months' follow-up using the Mini International Neuropsychiatric Interview questionnaire (MINI 5.0.0). PWID medical history, drug use, and sociodemographic and clinical characteristics were also collected. Among 188 PWID who participated in the assessment, 48 (25.5%) had at least one psychiatric disorder and 19 (10.1%) had 2 or more psychiatric disorders. The most common current psychiatric disorders were major depressive episode (12.2%) and psychotic disorder (4.8%), reaching 10.1% for the latter when lifetime prevalence was considered. Females were more likely than males to have at least one psychiatric disorder, a major depressive disorder, or an anxiety disorder. Methamphetamine use was associated with an increased risk of presenting a lifetime psychotic syndrome. Problematic alcohol consumption was associated with an increased risk of having at least one psychiatric disorder. Psychiatric comorbidities are frequent among PWID in Vietnam. These results highlight the need for routine assessment and innovative interventions to address mental health needs among PWID. Community-based interventions targeting mental health prevention and care should be strongly supported.

## 1. Introduction

People who use drugs are particularly susceptible to mental health disorders [1–3], the most common being mood and anxiety disorders, suicidal ideation, and suicide attempt [2, 4]. Among people who inject drugs (PWID), the prevalence of mental disorders can reach 40% and increases significantly if the individual has a lifetime prevalence of mental disorders [2]. Determinants of psychiatric disorders among PWID may include social, environmental, psychological, and medical factors and may differ based on the type of substance used. HIV/HCV seropositivity, gender (female), trauma during childhood, stigma due to drug use, and related conditions and excessive alcohol use are commonly associated with depression. Stimulant use is particularly associated with psychotic syndrome [5–8]. Drug users suffering from mental health disorders are more likely to engage in high-risk behaviors and have negative outcomes for the treatment of HIV infection and substance use disorders [9]. Stimulant use is particularly associated with psychiatric complications, especially with psychotic symptoms [10], but can also be associated with sexual or injection-related risk behavior and delayed access to methadone initiation for opiate dependency [11, 12].

HIV infection is common among PWID, and people suffering from mental health disorders may delay access to antiretroviral treatment (ART) and have poor virologic response to first-line ART, have low adherence to treatment and impaired quality of life [13, 14], and may exhibit increased prevalence of sexual related risk behaviors [15]. Currently, one major recommendation for addressing mental health is to plan a comprehensive, multidisciplinary, integrated approach to treatment, including drug use and mental health assessment and care for at-risk populations, including PWID [9, 16].

The overall prevalence of psychiatric disorders among PWID in Asia is comparable to Western countries [2]. A major concern in Asia, however, is the increasing use of methamphetamine in the most vulnerable populations (PWID, female sex workers, men who have sex with men) which is associated with several HIV risk-related behaviors [17, 18]. In Singapore, the mean annual total cost of managing a depressive patient was as high as $7,638 USD, in which the indirect costs (81%) dominated the total costs [19]. As in many low-to-middle income countries [16, 20], psychiatric resources are limited in Vietnam, and even with health insurance coverage, access to mental health treatment is generally complex and expensive. Additionally, there are very few therapeutic options [21, 22]. The stigmatization of mental illness thus constitutes a significant barrier for access to care.

Hai Phong is the largest harbor city in North Vietnam with approximately 2 million inhabitants. It has an estimated population of 5,000 PWID [23] with 3,955 patients treated with methadone in March 2017 (Vietnamese Ministry of Health). It has documented a very high prevalence of HIV among PWID, reaching 66% in 2006, decreasing to 48% in 2009 and 30% in 2016. As a result of “combined prevention” programs including increased access to ART, methadone treatment, and harm reduction interventions (mainly information-education-communication, needle/syringe exchange programs, and condoms), the estimated HIV incidence among PWID is now 0.8/100 person-years [24]. There is little information available on the prevalence of mental disorders and access to psychiatric care among PWID and as doctors in methadone and HIV clinics are not allowed to prescribe psychotropic drugs, prescriptions are highly restricted and only dispensed by registered clinical psychiatrists.

In the period of September 2014 to September 2016, the DRIVE-IN study (Drugs and Viral Infections in Vietnam–Initial Phase) was carried out in Hai Phong. The primary objective of the DRIVE-IN project was to evaluate the feasibility of implementing an interventional cohort of PWID in Hai Phong to demonstrate that the enrolment and follow-up of various hard-to-reach subgroups of PWID were feasible in the local context. The objective of the second phase (the DRIVE project), through a large scale intervention, is to show that we can get close to zero HIV incidence among PWID in Hai Phong. In this study, we present the results carried out at week 64 among DRIVE-IN PWID cohort participants.

## 2. Materials and Methods

### 2.1. Study Design

The current cross-sectional assessment of psychiatric comorbidities was conducted among a sample of PWID included in the DRIVE-IN cohort in Hai Phong, Vietnam [12, 24, 25]. The psychiatric assessment took place during the last visit of the cohort follow-up (the week 64 visit), from March 14 to May 6, 2016.

### 2.2. Recruitment of Study Subjects

Two hundred fifty subjects were included in the DRIVE-IN cohort (for more details, see Michel, Des Jarlais [12]); they were active PWID (verified by skin marks and a positive urine test for opiates or methamphetamine), 18 years of age or older, and capable of giving informed consent. Follow-up was conducted at 19, 32, 52, and 64 weeks; visits took place in a community-based organization (CBO, peer support group) office in Hai Phong City. This psychiatric assessment was systematically offered to all participants of the DRIVE-IN cohort who came to the week 64 visit.

### 2.3. Data Collection

Data was collected using a face-to-face questionnaire covering drug use, psychiatric disorders, and use of services. HIV and HCV testing along with urine testing for drug detection (Nal Von Minden Drug-Screen Urine® rapid test) were conducted during the visit.

The questionnaire was based on instruments previously used by the research team, assessing patterns of drug use, comorbidities, access to MMT and ART, suicide attempt, overdose, and social situation (household registration, medical insurance) with adaptation and pretesting for use in Hai Phong. The drug use questions covered the three months prior to the interview. Hazardous and binge drinking were assessed using the Audit-C questionnaire [26, 27].

### 2.4. Psychiatric Assessment

Current and lifetime psychiatric and substance use disorders were screened for by trained doctors from Hai Phong University of Medicine and Pharmacy using the Mini International Neuropsychiatric Interview (MINI), which is a structured diagnostic psychiatric interview based on the Diagnostic and Statistical Manual of Mental Disorders criteria, 4th Edition (DSM-IV) [28]. This interview recorded information on major depressive episodes, dysthymia, suicidality, (hypo)manic episodes, panic disorder, agoraphobia, social phobia, obsessive-compulsive disorder, post-traumatic stress disorder (PTSD), generalized anxiety disorder (GAD), psychotic disorder, and substance use disorders (i.e., alcohol and drug abuse/dependence). The MINI questionnaire was translated into Vietnamese following a translation–back translation–pre-test strategy [29–31]. Patients who screened positive for any mental health diagnoses were reported to the psychiatric staff in the Mental Health Department of Hai Phong University of Medicine and Pharmacy, and an appointment was offered with a psychiatrist, first for a clinical assessment in the CBO office where the study took place, and later in the mental health department if a follow-up was necessary. Participants requiring treatment received a free medical prescription from a psychiatrist.

Participants were compensated (150,000 VND–$7.50 USD) for their participation in the study and for transportation expenses.

All interviews and data collection were performed by medical staff (doctors and nurses) of Hai Phong University of Medicine and Pharmacy who had been trained by research coordinators (international and national experts from different fields).

### 2.5. Data Management and Analysis

All data was collected on an electronic case report form using ClinCapture® software which complies with the FDA clinical trial recommendations (21 CFR part 11). Analyses were performed using Stata® v10 (Stata Corp, College Station, TX, USA).

For quantitative variables, the means and standard deviations or the median with the interquartile range are presented. For the qualitative variables, frequencies and percentages are presented.

In statistical analysis, mental health problems were divided into four types of psychiatric disorder (nonexclusive): at least one current psychiatric disorder captured by the MINI questionnaire (major depressive episode, dysthymia, etc.), current major depressive disorder, any current anxiety disorder, and lifetime psychotic syndrome.

We analyzed the risk factors for each type of disorder using multivariable logistic regression. Specifically, we first analyzed risk factors in a univariable analysis. The risk factors taken into account included sociodemographic characteristics, comorbidities, and drug and alcohol use. All factors with a p<0.2 were retained for the multivariable analysis using backward elimination. The goodness of fit was assessed using the Hosmer-Lemeshow test. P-values below 0.05 were considered statistically significant. The model was also tested for multicollinearity by checking the correlation matrix of coefficients of regression and calculating variance inflation factors.

### 2.6. Institutional Review Board Approval

The study was approved by the Institutional Review Boards of the Hai Phong University of Medicine and Pharmacy, Vietnam (No. 05/HDDD), and the Icahn School of Medicine, Mount Sinai, United States (No. MSBI-100-14). All participants signed a written informed consent.

## 3. Results

Among the 250 PWID who gave their consent for a 64-week follow-up, 194 participants came back for a visit during week 64 and 188 agreed to a psychiatric assessment. Their main characteristics are presented in Table 1. 118 (62.8%) were currently being treated with methadone (*versus* 11 (4.4%) at cohort initiation), 76 (40.4%) self-reported smoking methamphetamine (92 (48.9%) on the basis of self-report and urine test), and 90 were poly drug users (47.9%), the most common being dual heroin and methamphetamine use. Compared to those who participated in the psychiatric assessment, the 62/250 participants who dropped out from the follow-up or refused the psychiatric assessment were not significantly different with respect to age (mean age of 38.3 versus 38.4, p=0.452), gender (male: 88.7% versus 87.2%, p=0.760), monthly income (mean 4,450,000 versus 4.500.000 VND, p=0.153), living in couple (30.7% versus 36.7%, p=0.387), or injection years (mean 8,7 versus 8.9, p= 0.983). Nine PWID died during follow-up, including 2 from overdose and 2 from suicide.

### 3.1. Psychiatric Disorders

Table 2 presents the frequency of psychiatric disorders according to the MINI questionnaire at week 64: 48/188 (25.5%) had at least one current psychiatric disorder and 19 (10.1%) had 2 or more psychiatric disorders. The most common current psychiatric disorders were major depressive episode (12.2%) and psychotic disorder (4.8%), reaching 10.1% for the latter when lifetime prevalence was taken into account. Among female sex workers, 10/21 (47.6%) presented at least one psychiatric disorder, 9/21 (42.9%) a major depressive disorder, and 6/21 (28.6%) at least one anxiety disorder.

### 3.2. Clinical Psychiatric Assessment

The 48 participants who screened positive for a psychiatric disorder, including 14 in a serious clinical situation (high suicide risk and/or psychotic syndrome), were offered a clinical assessment with a psychiatrist at the study site; 39 (81%) agreed and met the psychiatrist (including 12 of the 14 participants in a serious clinical situation) who carried out a full psychiatric diagnosis. Of the 39 who met with the psychiatrist, none of the PWID came back subsequently to the mental health department for hospitalization or an outpatient consultation.

### 3.3. Factors Associated with Having a Psychiatric Disorder

Tables 3–6, respectively, present the results of univariable and multivariable analyses for factors associated with having any psychiatric disorder (Table 3), having a major depressive disorder (Table 4), having any anxiety disorder (Table 5), or having a lifetime psychotic syndrome (Table 6). For each model, independent variables were checked for multicollinearity, of which all variance inflation factors were < 5.

The various multivariable analyses showed that females were more likely than males to have at least one psychiatric disorder (Adjusted Odds Ratio (AOR): 3.03, 95%CI: 1.02-9.03), a major depressive disorder (AOR[95%CI]: 5.05[1.54-16.60]), or an anxiety disorder (AOR[95%CI]: 3.14[1.04-9.49]). Methamphetamine use was associated with an increased risk of presenting a lifetime psychotic syndrome (AOR[95%CI]: 4.05[1.25-13.15]). Problematic alcohol consumption was associated with an increased risk of having at least one psychiatric disorder (AOR[95%CI]: 2.53[1.19-5.37]).

## 4. Discussion

Using the MINI questionnaire, based on the DSM-IV diagnosis criteria, we found that approximately 25% of the PWID had at least one current psychiatric disorder at the time of the study. The rate of psychiatric comorbidities among the participants in our study sample was high, but lower than the prevalence observed in other PWID populations [2] including China [32, 33], Italy [34], the Netherlands [35], and the United States [36]. The current and lifetime depression rates in our study were comparable to community rates reported by meta-analysis results from 30 countries between 1994 and 2014 [37].

There are several plausible explanations for the results of our study. First, PWID were not recruited among subjects seeking care and thus were more likely to be prone to psychiatric disorders [4, 38]; they were assessed at week 64 and not at cohort initiation, and a large number were receiving methadone treatment that was initiated during follow-up along with support from their CBO-peers, including counseling, harm reduction interventions, support in accessing health insurance, and housing. Second, there was heterogeneity in the methodologies (diagnostic criteria, assessment tools, and population sample selection) used in the literature to assess the prevalence of psychiatric disorders [8]. In our survey, only Axis 1 psychiatric disorders on the MINI questionnaire (mood disorders, anxiety disorder, and psychotic syndrome) were taken into account to assess the overall prevalence of psychiatric disorders, which would exclude particular disorders, such as personality disorders.

Given the great burden of mental health problems among PWID populations, and the association between mental health problems, increased risk-related behaviors, and decreased effectiveness in terms of prevention, access to care, and quality of life, screening and care for mental health pathologies should be integrated into drug use and HIV treatment clinics as part of the regular patient intake process.

Our findings highlight the great need for innovative forms of psychiatric care for PWID in Vietnam, particularly with provision of care in the community, and the need to further examine factors associated with failure to attend services. In addition, the factors associated with presenting a psychiatric disorder in this study highlight the crucial role of methamphetamine and alcohol use, and also the vulnerability of female PWID.

Being female is associated with an increased prevalence of psychiatric disorders, including major depressive disorder and anxiety disorder. A higher prevalence of psychiatric disorders among women compared to men is well documented, particularly for depressive and anxiety disorders [39, 40]. The fact that 21 of the 24 women in our cohort were sex workers could explain this result, as mental distress is frequently documented among sex workers [41, 42].

Methamphetamine use is associated with lifetime psychotic syndrome. The use of psycho-stimulants is also commonly associated with a substantial burden of psychopathology, which includes elevated rates of psychosis, mood, and anxiety disorders [43, 44]. One of the key clinical differences between psycho-stimulant and opioid use is that psycho-stimulant use can induce psychotic episodes. Case-control studies have reported that psycho-stimulant users have higher levels of psychotic disorders than opioid, benzodiazepine, and barbiturate users [45–48]. Methamphetamine use in recent years has increased markedly in Vietnam [12, 17]. However the majority of drug users are not aware of the risks associated with using amphetamine-type stimulants [17, 25]. This is a major concern for the Vietnamese healthcare and social system for health education, harm reduction, HIV, HCV, and mental health prevention and care in this population of drug users [12, 17, 25].

Problematic alcohol consumption was associated with having at least one psychiatric disorder. Alcohol abuse can cause depression, anxiety, psychosis, and antisocial behavior, both during intoxication and during withdrawal [8, 49, 50]. When alcohol use disorders and psychiatric disorders cooccur, patients are more likely to have difficulty maintaining abstinence, to attempt or commit suicide, and to use health services [51–53]. Thus, the implementation of mental healthcare services for PWID should be carried out in conjunction with care for abuse of other substances such as methamphetamine and should include alcohol abuse screening and management. Given the inadequate coverage of general mental health services in the community in Vietnam, in a field where human resources are insufficiently trained [54], a specific training program for health workers in the health care system is urgently needed.

PWID in the study frequently agreed to meet a psychiatrist in community-based premises but were reluctant to go to a psychiatric hospital, even for an outpatient consultation; in fact, none of the PWID came back for treatment at the psychiatric hospital or for an outpatient consultation. This general reluctance indicates a need to develop outreach psychiatric care within the community to reduce stigma associated with seeking mental health services.

There are several implications from this study. First, screening for mental health problems should be routinely implemented, particularly among high-risk populations such as PWID. This is a complex issue in Vietnam, where access to psychiatric care is limited, psychiatric disorders and drug use are stigmatized, and there is a lack of specialized knowledge among professionals in methadone or HIV clinics to handle mental health problems. Routine assessments and dedicated routes of access to care should be organized according to local resources, including integrated care in MMT clinics and CBO support [12, 53, 55]. Recent data has shown the ability of community-based organizations to screen for and identify disorders, maintain contact, and provide harm reduction tools for PWID and to support PWID in accessing methadone treatment, particularly by overcoming administrative barriers [12, 25, 56]. In countries where the scaling-up of health services dedicated to addiction medicine cannot cover the needs of PWID, CBO interventions should be strongly supported, as they can mobilize quickly and reach this population easily.

There are several limitations to this study that should be noted. First, we used a cross-sectional design, and as a result we could not establish temporal relationships across variables. Psychiatric problems can occur before or after the onset of a drug addiction; certainly, this chronological order can have consequences on the approach to treatment. Second, because we used a convenience sample, we cannot generalize our findings to the PWID population as a whole. In addition, our small sample size reduced the statistical power of our study. The association between HIV status and depression, for example, common in the literature [57], could have reached significance with a larger sample size; these findings should therefore be replicated in a larger sample of PWID. Finally, some significant and frequent comorbidities such as attention deficit and hyperactivity disorder [58] or medical complications associated with intravenous drug use such as infective endocarditis [59] and cutaneous complications [60] were not reported in our study, and as a result, we were unable to examine associations with respect to these variables.

## 5. Conclusion

The findings presented in this study confirm that there are frequent psychiatric comorbidities among PWID in Vietnam. Many factors, including gender, methamphetamine use, and problematic alcohol consumption, appear linked to mental health disorders. These results highlight the need for routine assessment and adaptation of the mental health system to address the needs of PWID. CBO interventions should be strongly supported to screen for and identify disorders, maintain contact, and provide access to care by overcoming administrative barriers.

## Figures and Tables

**Table 1:** Sociodemographic and clinical characteristics among PWID in Hai Phong, Vietnam (N=188).

	**N (**%**)**
***Sociodemographics***	
Gender	
Male	164 (87.2)
Female	24 (12.8)
Age (Mean, SD)	38.4 (8.4)
Living with a partner	
Yes	69 (36.7)
No	119 (63.3)
Any children	
Yes	113 (60.1)
No	75 (39.9)
Monthly income (VND) (Median, IQR)	3,500,000 [3,000,000-5,000,000]
Medical Insurance in Hai Phong	
Yes	159 (84.6)
No	29 (15.4)
Household Registration in Haiphong	
Yes	179 (95.2)
No	9 (4.8)
***Drug data***	
Urine toxicology	
Benzodiazepine	13 (6.9)
Methamphetamine (“Ice”)	77 (41.0)
Heroin	135 (71.8)
Methadone	120 (63.8)
Drug use (self-report)	
Injected drugs	135 (71.8)
Heroin	135 (100)
Other drugs	0 (0)
Non-injected drugs	
Heroin	16 (8.5)
Methamphetamine (“Ice”)	76 (40.4)
Other	22 (11.7)
Poly drug use (urine test)	90 (47.9)
Methamphetamine (“Ice”) use (self-report and urine test)	92 (48.9)
Number of years of injection (Mean, SD)	8.9 (6.6)
Duration of injection use (N,%)	
Less than 5 years	71 (38.0)
Greater than or equal to 5 years	116 (62.0)
Number of injections in a typical day (last 30 days) (Mean, SD)	1.2 (1.2)
Alcohol use (Audit-C)	
Hazardous use	55 (29,3)
Binge drinking	20 (10.6)
Methadone treatment (self-report)	118 (62.8)
Serology	
HIV positive	36 (19.2)
HCV positive	119 (63.3)
Past history of overdose	34 (18.1)
Past history of suicide attempt	26 (13.8)

**Table 2:** Frequency of psychiatric disorders among PWID in Hai Phong, Vietnam, according to the MINI questionnaire (N=188).

	**N (**%**)**	
At least one psychiatric disorder^&^	48 (25.5)	
Number of psychiatric disorders (N=48)^&^		
1	29 (15.4)	
2	10 (5.3)	
3	5 (2.7)	
4	0 (0)	
5	3 (1.6)	
6	1 (0.5)	
Mood disorder^&^ (any)	27 (14.4)	
Anxiety disorder^&^ (any)	28 (14.9)	
	**Current**	**Lifetime**
Major depressive episode	23 (12.2)	9 (4.8)
Dysthymia	5 (2.7)	
Suicidality		
Suicide risk (any level)	36 (19.1)	
Low risk	25 (13.3)	
Moderate risk	5 (2.7)	
High risk	6 (3.2)	
(Hypo)manic episode	8 (4.3)	18 (9.6)
Panic disorder	4 (2.1)	11 (5.9)
Agoraphobia	10 (5.3)	
Social phobia	3 (1.6)	
Obsessive-compulsive disorder	4 (2.1)	
Post-traumatic stress disorder	6 (3.2)	
Generalized anxiety disorder	9 (4.8)	
Psychotic disorder	13 (6.9)	19 (10.1)
Alcohol use disorder		
Abuse	8 (4.3)	
Dependence	3 (1.6)	
Drug use disorder		
Dependence*∗*	148 (78.7)	
Heroin	145 (77.1)	
Methamphetamine (“Ice”)	11 (5.9)	
Other*∗∗*	3 (1.6)	
Abuse*∗*	39 (20.1)	
Heroin	10 (5.3)	
Methamphetamine (“Ice”)	29 (15.4)	
Other*∗∗*	3 (1.6)	

^&^Only current disorders.

*∗*Subjects can be dependent/abusers with several drugs at the same time.

*∗∗*Other amphetamines, cannabis, methadone.

**Table 3:** Factors associated with having at least one psychiatric disorder among PWID in Hai Phong, Vietnam (N=188).

Variables		At least one psychiatric disorder N (%)	OR (95% CI)	p value	Adjusted OR (95% CI)	p value
Sex	Male/Trans	36 (22.0)	REF		REF	
	Female	12 (50.0)	3.56 (1.47- 8.59)	**0.005**	3.03 (1.02-9.03)	**0.046**
Age	≤ 35	21 (27.6)	REF		REF	
	> 35	27 (24.1)	0.83 (0.42- 1.61)	0.587	-	-
Marital status	Married/partner	18 (26.1)	REF		REF	
	Other	30 (25.2)	0.95 (0.48- 1.88)	0.894	-	-
Children	No children	17 (22.7)	REF		REF	
	Has Children	31 (27.4)	1.28 (0.65- 2.54)	0.464	-	-
Monthly income_ _^*∗*^	≤ 3M VND	19 (20.9)	REF		REF	
	> 3M VND	29 (29.9)	1.61 (0.82- 3.14)	0.158	1.23 (0.59-2.57)	0.581
Illness related to HIV (last 3 months)	No	45 (25.0)	REF		REF	
	Yes	3 (37.5)	1.80 (0.41- 7.73)	0.433	-	-
Past history of overdose	No	37 (24.0)	REF		REF	
	Yes	11 (32.4)	1.51 (0.67- 3.39)	0.316	-	-
No. of years of injection	≤8 years	24 (23.3)	REF		REF	
	>8 years	23 (27.4)	1.24 (0.64- 2.40)	0.523	-	-
Urine toxicology: benzodiazepine	No	45 (25.7)	REF		REF	
	Yes	3 (23.1)	0.87 (0.23- 3.29)	0.833	-	-
Urine toxicology: heroin	No	14 (26.4)	REF		REF	
	Yes	34 (25.2)	0.93 (0.45- 1.93)	0.862	-	-
Urine toxicology: methadone	No	17 (25.0)	REF		REF	
	Yes	31 (25.8)	1.04 (0.52- 2.07)	0.900	-	-
Number of injections per month	<75	39 (25.0)	REF		REF	
	≥ 75	9 (28.1)	1.17 (0.50- 2.75)	0.712	-	-
Problematic alcohol use (Audit-C)^&^	No	29 (21.8)	REF		REF	
	Yes	19 (34.6)	1.89 (0.94- 3.78)	0.071	2.53 (1.19-5.37)	**0.016**
Risk-related sexual practices	No	45 (25.6)	REF		REF	
	Yes	3 (25.0)	0.97 (0.25- 3.74)	0.965	-	-
Methamphetamine (“Ice”) use^0^	No	19 (19.8)	REF		REF	
	Yes	29 (31.5)	1.86 (0.95- 3.63)	0.067	1.76 (0.87-3.57)	0.117
Still on methadone at W64 visit	No	3 (23.1)	REF		REF	
	Yes	29 (24.6)	1.08 (0.27- 4.21)	0.905	-	-
Currently receiving ART at W64 visit	No	40 (24.4)	REF		REF	
	Yes	8 (33.3)	1.55 (0.62-3.89)	0.351	-	-
HIV status	Negative	35 (23.0)	REF		REF	
	Positive	13 (36.1)	1.88 (0.86- 4.11)	0.109	1.71 (0.72-4.08)	0.227
HCV status	Negative	20 (29.0)	REF		REF	
	Positive	28 (23.5)	0.75 (0.38- 1.47)	0.409	-	-

**∗** VND: Vietnam Dong (≤ or > 3 000 000 VND).

^&^ Audit-c score ≥3 in women and ≥4 in men.

°Self-reported methamphetamine use + urine test.

**Table 4:** Factors associated with having a major depressive disorder (current) among PWID in Hai Phong, Vietnam (N=188).

Variables		Major depressive disorder N (%)	OR (95% CI)	p value	Adjusted OR (95% CI)	p value
Sex	Male/Trans	13 (7.9)	REF		REF	
	Female	10 (41.7)	8.29 (3.08- 22.30)	**<0.001**	5.05 (1.54-16.60)	**0.007**
Age	≤ 35	12 (15.8)	REF		REF	
	> 35	11 (9.8)	0.58 (0.24- 1.39)	0.224	-	-
Marital status	Married/partner	7 (10.1)	REF		REF	
	Other	16 (13.5)	1.37 (0.53- 3.53)	0.507	-	-
Children	No children	9 (12.0)	REF		REF	
	Has Children	14 (12.4)	1.03 (0.42- 2.53)	0.936	-	-
Monthly income_ _^*∗*^	≤ 3M VND	9 (9.9)	REF		REF	
	> 3M VND	14 (14.4)	1.53 (0.63- 3.74)	0.345	-	-
Illness related to HIV (last 3 months)	No	20 (11.1)	REF		REF	
	Yes	3 (37.5)	4.8 (1.06- 21.61)	**0.041**	1.71 (2.23-12.82)	0.603
Past history of overdose	No	17 (17.0)	REF		REF	
	Yes	6 (17.6)	1.72 (0.62- 4.76)	0.292	-	-
No. of years of injection	≤8 years	11 (10.7)	REF		REF	
	>8 years	11 (13.1)	1.26 (0.51- 3.07)	0.611	-	-
Urine toxicology: benzodiazepines	No	22 (12.6)	REF		REF	
	Yes	1 (7.7)	0.58 (0.07-4.68)	0.609	-	-
Urine toxicology: heroin	No	9 (17.0)	REF		REF	
	Yes	14 (10.4)	0.56 (0.23- 1.40)	0.218	-	-
Urine toxicology: methadone	No	8 (11.7)	REF		REF	
	Yes	15 (12.5)	1.07 (0.43- 2.67)	0.882	-	-
Number of injections per month	<75	19 (12.2)	REF		REF	
	≥ 75	4 (12.5)	1.03 (0.32- 3.26)	0.960	-	-
Problematic alcohol use (Audit-C)^&^	No	15 (11.3)	REF		REF	
	Yes	8 (14.6)	1.34 (0.53- 3.36)	0.535	-	-
Methamphetamine (“Ice”) use^0^	No	12 (12.5)	REF		REF	
	Yes	11 (12.0)	0.95 (0.40-2.27)	0.909	-	-
Still on methadone at W64 visit	No	3 (23.1)	REF		REF	
	Yes	14 (11.9)	0.45 (0.11- 1.83)	0.264	-	-
Currently receiving ART at W64 visit	No	20 (12.2)	REF		REF	
	Yes	3 (12.5)	1.03 (0.28-3.76)	0.966	-	-
HIV status	Negative	16 (10.5)	REF		REF	
	Positive	7 (19.4)	2.05 (0.77- 5.43)	0.148	1.05 (0.29-3.81)	0.940
HCV status	Negative	11 (15.9)	REF		REF	
	Positive	12 (10.1)	0.59 (0.24- 1.42)	0.241	-	-

**∗** VND: Vietnam Dong (≤ or > 3 000 000 VND).

^&^ Audit-c score ≥3 in women and ≥4 in men.

°Self-reported methamphetamine use + urine test.

**Table 5:** Factors associated with having an anxiety disorder (current) among PWID in Hai Phong, Vietnam (N=188).

Variables		Any anxiety disorder N (%)	OR (95% CI)	p value	Adjusted OR (95% CI)	p value
Sex	Male/Trans	20 (12.2)	REF		REF	
	Female	8 (33.3)	3.6 (1.36- 9.48)	**0.009**	3.14 (1.04-9.49)	**0.043**
Age	≤ 35	13 (17.1)	REF		REF	
	> 35	15 (13.4)	0.75 (0.33- 1.68)	0.484	-	-
Marital status	Married/couple	11 (15.9)	REF		REF	
	Other	17 (14.3)	0.88 (0.38- 2.00)	0.759	-	-
Children	No children	8 (10.7)	REF		REF	
	Has Children	20 (17.7)	1.8 (0.75- 4.33)	0.189	1.56 (0.62-3.95)	0.345
Monthly income_ _^*∗*^	≤ 3M VND	11 (12.1)	REF		REF	
	> 3M VND	17 (17.5)	1.54 (0.68- 3.50)	0.298	-	-
Illness related to HIV (last 3 months)	No	26 (14.4)	REF		REF	
	Yes	2 (25.0)	1.97 (0.38- 10.31)	0.420	-	-
No. of years of injection	≤8 years	16 (15.5)	REF		REF	
	>8 years	11 (13.1)	0.81 (0.35- 1.87)	0.637	-	-
Urine toxicology: benzodiazepines	No	26 (14.9)	REF		REF	
	Yes	2 (15.4)	1.04 (0.21- 4.97)	0.959	-	-
Urine toxicology: heroin	No	8 (15.1)	REF		REF	
	Yes	20 (14.8)	0.97 (0.40- 2.38)	0.961	-	-
Urine toxicology: methadone	No	12 (17.7)	REF		REF	
	Yes	16 (13.3)	0.71 (0.31- 1.62)	0.426	-	-
Number of injections per month	<75	23 (14.7)	REF		REF	
	≥ 75	5 (15.6)	1.07 (0.37- 3.06)	0.899	-	-
Problematic alcohol use(Audit-C)^&^	No	19 (14.3)	REF		REF	
	Yes	9 (16.4)	1.17 (0.49- 2.78)	0.716	-	
Methamphetamine (“Ice”) use^0^	No	9 (9.4)	REF		REF	
	Yes	19 (20.7)	2.51 (1.07- 5.89)	**0.034**	2.28 (0.94-5.47)	0.064
Still on methadone at W64 visit	No	2 (15.4)	REF		REF	
	Yes	16 (13.6)	0.86 (0.17- 4.25)	0.865	-	-
Currently receiving ART at W64 visit	No	25 (15.2)	REF		REF	
	Yes	3 (12.5)	0.79 (0.22-2.86)	0.724	-	-
HIV status	Negative	22 (14.5)	REF		REF	
	Positive	6 (16.7)	1.18 (0.44- 3.16)	0.740	-	-
HCV status	Negative	14 (20.3)	REF		REF	
	Positive	14 (11.8)	0.52 (0.23- 1.17)	0.117	0.48 (0.21-1.12)	0.088

**∗** VND: Vietnam Dong (≤ or > 3 000 000 VND).

^&^ Audit-c score ≥3 in women and ≥4 in men.

°Self-reported methamphetamine use + urine test.

**Table 6:** Factors associated with having a psychotic syndrome (lifetime) among PWID in Hai Phong, Vietnam (N=188).

Variables		Psychotic syndrome N (%)	OR (95% CI)	p value	Adjusted OR (95% CI)	p value
Sex	Male/Trans	15 (9.2)	REF		REF	
	Female	4 (16.7)	1.98 (0.60-6.58)	0.261	-	-
Age	≤ 35	11 (14.5)	REF		REF	
	> 35	8 (7.1)	0.45 (0.17-1.18)	0.108	0.45 (0.16-1.29)	0.135
Marital status	Married/partner	6 (8.7)	REF		REF	
	Other	13 (10.9)	1.28 (0.47-3.56)	0.626	- -	-
Children	No children	9 (12.0)	REF		REF	
	Has Children	10 (8.9)	0.71 (0.27-1.84)	0.484	-	-
Monthly income_ _^*∗*^	≤ 3M VND	10 (11.0)	REF		REF	
	> 3M VND	9 (9.3)	0.83 (0.32-2.14)	0.698	-	-
Illness related to HIV (last 3 months)	No	19 (10.6)	REF		REF	
	Yes	0	-	-	-	-
Past history of overdose	No	16 (10.4)	REF		REF	
	Yes	3 (8.8)	0.83 (0.23-3.04)	0.784	-	-
No. of years of injection	≤8 years	12 (11.7)	REF		REF	
	>8 years	7 (8.3)	0.69 (0.26-1.83)	0.457	-	-
Urine toxicology: benzodiazepines	No	16 (9.1)	REF		REF	
	Yes	3 (23.1)	2.98 (0.74-11.9)	0.123	0.10 (0.16-3.91)	0.780
Urine toxicology: heroin	No	4 (7.6)	REF		REF	
	Yes	15 (11.1)	1.53 (0.48-4.84)	0.72	-	-
Urine toxicology: methadone	No	9 (13.2)	REF		REF	
	Yes	10 (8.3)	0.59 (0.23-1.54)	0.288	-	-
Number of injections per month	<75	16 (10.3)	REF		REF	
	≥ 75	3 (9.4)	0.91 (0.25-3.30)	0.880	-	-
Problematic alcohol use (Audit-C)^&^	No	13 (9.8)	REF		REF	
	Yes	6 (10.9)	1.13 (0.40-3.14)	0.814	-	-
Methamphetamine (“Ice”) use^0^	No	4 (4.2)	REF		REF	
	Yes	15 (16.3)	4.48 (1.42-14.06)	**0.008**	4.05 (1.25-13.15)	**0.020**
Still on methadone at W64 visit	No	2 (15.4)	REF		REF	
	Yes	11 (9.3)	0.56 (0.11-2.88)	0.493	-	-
Currently receiving ART at W64 visit	No	16 (9.8)	REF		REF	
	Yes	3 (12.5)	1.32 (0.36-4.92)	0.677	-	-
HIV status	Negative	16 (10.5)	REF		REF	
	Positive	3 (8.3)	0.77 (0.21-2.81)	0.695	-	-
HCV status	Negative	11 (15.9)	REF		REF	
	Positive	8 (6.7)	0.38 (0.14-0.99)	**0.049**	0.41 (0.16-0.29)	0.091

**∗** VND: Vietnam Dong (≤ or > 3 000 000 VND).

^&^ Audit-c score ≥3 in women and ≥4 in men.

°Self-reported methamphetamine use + urine test.

## Data Availability

The EXCEL/STATA data used to support the findings of this study are available from the corresponding author upon request.

## Abbreviations

ANRS: French Agence Nationale de Recherches sur le Sida et les hépatites virales
ART: Antiretroviral treatment
CBO: Community-based organization (peer support group)
CFR part 11: Part 11 of the Code of Federal Regulations of the US FDA on electronic records and electronic signatures
DSM-IV: Diagnostic and Statistical Manual of Mental Disorders, 4th Edition
FDA: Food and Drug Administration, United States
GAD: Generalized anxiety disorder
HIV: Human immunodeficiency virus
HCV: Hepatitis C Virus
MINI: Mini International Neuropsychiatric Interview
MMT: Methadone maintenance therapy
MSM: Men having sex with men
PTSD: Post-traumatic stress disorder
PWID: People who inject drugs
VND: Vietnam Dong (Vietnamese Currency)
USD: US Dollar.

## References

[1] Hasin D. S., Grant B. F. (2015). The National Epidemiologic Survey on Alcohol and Related Conditions (NESARC) Waves 1 and 2: review and summary of findings. *Social Psychiatry and Psychiatric Epidemiology*.

[2] Iskandar S., Kamal R., De Jong C. A. (2012). Psychiatric comorbidity in injecting drug users in Asia and Africa. *Current Opinion in Psychiatry*.

[3] Torrens M., Gilchrist G., Domingo-Salvany A. (2011). Psychiatric comorbidity in illicit drug users: Substance-induced versus independent disorders. *Drug and Alcohol Dependence*.

[4] Compton W. M., Thomas Y. F., Stinson F. S., Grant B. F. (2007). Prevalence, correlates, disability, and comorbidity of DSM-IV drug abuse and dependence in the United States: Results from the national epidemiologic survey on alcohol and related conditions. *Archives of General Psychiatry*.

[5] EMCDDA (2015). *Comorbidity of substance use and mental disorders in Europe*.

[6] Davis L., Uezato A., Newell J. M., Frazier E. (2008). Major depression and comorbid substance use disorders. *Current Opinion in Psychiatry*.

[7] Levintow S. N., Pence B. W., Ha T. V. (2018). Prevalence and predictors of depressive symptoms among HIV-positive men who inject drugs in Vietnam. *PLoS ONE*.

[8] Lai H. M. X., Cleary M., Sitharthan T., Hunt G. E. (2015). Prevalence of comorbid substance use, anxiety and mood disorders in epidemiological surveys, 1990-2014: A systematic review and meta-analysis. *Drug and Alcohol Dependence*.

[9] Altice F. L., Kamarulzaman A., Soriano V. V., Schechter M., Friedland G. H. (2010). Treatment of medical, psychiatric, and substance-use comorbidities in people infected with HIV who use drugs. *The Lancet*.

[10] Salo R., Flower K., Kielstein A., Leamon M. H., Nordahl T. E., Galloway G. P. (2011). Psychiatric comorbidity in methamphetamine dependence. *Psychiatry Research*.

[11] Degenhardt L., Mathers B., Guarinieri M. (2010). Meth/amphetamine use and associated HIV: Implications for global policy and public health. *International Journal of Drug Policy*.

[12] Michel L., Des Jarlais D. C., Duong Thi H. (2017). Intravenous heroin use in Haiphong, Vietnam: Need for comprehensive care including methamphetamine use-related interventions. *Drug and Alcohol Dependence*.

[13] Pence B. W., Miller W. C., Gaynes B. N., Eron J. J. (2007). Psychiatric illness and virologic response in patients initiating highly active antiretroviral therapy. *Journal of Acquired Immune Deficiency Syndromes*.

[14] Ironson G., O'Cleirigh C., Fletcher M. A. (2005). Psychosocial factors predict CD4 and viral load change in men and women with human immunodeficiency virus in the era of highly active antiretroviral treatment. *Psychosomatic Medicine*.

[15] Kelly J. A., Murphy D. A., Bahr G. R. (1993). Factors Associated With Severity of Depression and High-Risk Sexual Behavior Among Persons Diagnosed With Human Immunodeficiency Virus (HIV) Infection. *Health Psychology*.

[16] Patel V., Chisholm D., Parikh R. (2016). Addressing the burden of mental, neurological, and substance use disorders: Key messages from Disease Control Priorities, 3rd edition. *The Lancet*.

[17] Vu N. T. T., Holt M., Phan H. T. T. (2016). Amphetamine-type stimulant use among men who have sex with men (MSM) in Vietnam: Results from a socio-ecological, community-based study. *Drug and Alcohol Dependence*.

[18] UNODC *Amphetamine type stimulants in Viet Nam: a review of the availability, use and implications for health and security in Viet Nam. Ha Noi, Viet Nam: United Nations Office on Drugs and Crime*.

[19] Ho R. C. M., Mak K.-K., Chua A. N. C., Ho C. S. H., Mak A. (2013). The effect of severity of depressive disorder on economic burden in a university hospital in Singapore.. *Expert review of pharmacoeconomics & outcomes research*.

[20] Eaton J., McCay L., Semrau M. (2011). Scale up of services for mental health in low-income and middle-income countries. *The Lancet*.

[21] Bao Giang K., Viet Dzung T., Kullgren G., Allebeck P. (2010). Prevalence of mental distress and use of health services in a rural district in Vietnam. *Global Health Action*.

[22] Niemi M., Thanh H. T., Tuan T., Falkenberg T. (2010). Mental health priorities in Vietnam: a mixed-methods analysis. *BMC Health Services Research*.

[23] Des Jarlais D., Khue P. M., Feelemyer J. (2018). Using dual capture/recapture studies to estimate the population size of persons who inject drugs (PWID) in the city of Hai Phong, Vietnam. *Drug and Alcohol Dependence*.

[24] Des Jarlais D. C., Thi Huong D., Thi Hai Oanh K. (2016). Prospects for ending the HIV epidemic among persons who inject drugs in Haiphong, Vietnam. *International Journal of Drug Policy*.

[25] Duong H. T., Jarlais D. D., Khuat O. H. (2018). Risk Behaviors for HIV and HCV Infection Among People Who Inject Drugs in Hai Phong, Viet Nam, 2014. *AIDS and Behavior*.

[26] Bush K., Kivlahan D. R., McDonell M. B., Fihn S. D., Bradley K. A. (1998). The AUDIT alcohol consumption questions (AUDIT-C): an effective brief screening test for problem drinking. *JAMA Internal Medicine*.

[27] Dawson D. A., Smith S. M., Saha T. D., Rubinsky A. D., Grant B. F. (2012). Comparative performance of the AUDIT-C in screening for DSM-IV and DSM-5 alcohol use disorders. *Drug and Alcohol Dependence*.

[28] Sheehan D. V., Lecrubier Y., Sheehan K. H. (1998). The mini-international neuropsychiatric interview (M.I.N.I.): the development and validation of a structured diagnostic psychiatric interview for DSM-IV and ICD-10. *Journal of Clinical Psychiatry*.

[29] Flaherty J. A., Gaviria F. M., Pathak D. (1988). Developing Instruments for Cross-Cultural Psychiatric Research. *The Journal of Nervous and Mental Disease*.

[30] Brislin R. W., Lonner W. J. B. (1986). *Field methods in Cross-Cultural research: Sage Publications*.

[31] Ghimire D. J., Chardoul S., Kessler R. C., Axinn W. G., Adhikari B. P. (2013). Modifying and validating the Composite International Diagnostic Interview (CIDI) for use in Nepal. *International Journal of Methods in Psychiatric Research*.

[32] Liao Y., Tang J., Liu T., Chen X., Liu X., Hao W. (2011). A pilot study of life events and mood disorders: Self-report survey in Chinese heroin-dependent individuals. *American Journal on Addictions*.

[33] Gu J., Lau J. T., Chen H., Chen X., Liu C., Liu J. (2010). Mental health and interpersonal factors associated with HIV-related risk behaviors among non-institutionalized female injection drug users who are also sex workers in China. *Women & health*.

[34] Maremmani A. G. I., Dell'Osso L., Pacini M. (2011). Dual diagnosis and chronology of illness in treatment-seeking italian patients dependent on heroin. *Journal of Addictive Diseases*.

[35] Carpentier P. J., Krabbe P. F. M., van Gogh M. T., Knapen L. J. M., Buitelaar J. K., de Jong C. A. J. (2009). Psychiatric comorbidity reduces quality of life in chronic methadone maintained patients. *American Journal on Addictions*.

[36] Jones D. L., Waldrop-Valverde D., Gonzalez P. (2010). Mental health in HIV seronegative and seropositive IDUs in South Florida. *AIDS Care*.

[37] Lim G. Y., Tam W. W., Lu Y., Ho C. S., Zhang M. W., Ho R. C. (2018). Prevalence of Depression in the Community from 30 Countries between 1994 and 2014. *Scientific Reports*.

[38] Grant B. F., Chou S. P., Goldstein R. B. (2008). Prevalence, correlates, disability, and comorbidity of DSM-IV borderline personality disorder: Results from the Wave 2 National Epidemiologic Survey on Alcohol and Related Conditions. *Journal of Clinical Psychiatry*.

[39] Steel Z., Marnane C., Iranpour C. (2014). The global prevalence of common mental disorders: a systematic review and meta-analysis 1980–2013. *International Journal of Epidemiology*.

[40] NPHN (2002). *National epidemiology mental disorder Survey by National Psychiatric Hospital No 1. Ho Chi Minh city, Vietnam*.

[41] Rössler W., Koch U., Lauber C. (2010). The mental health of female sex workers. *Acta Psychiatrica Scandinavica*.

[42] Lau J. T. F., Tsui H. Y., Ho S. P. Y., Wong E., Yang X. (2010). Prevalence of psychological problems and relationships with condom use and HIV prevention behaviors among Chinese female sex workers in Hong Kong. *AIDS Care*.

[43] Darke S., Kaye S., McKetin R., Duflou J. (2008). Major physical and psychological harms of methamphetamine use. *Drug and Alcohol Review*.

[44] Zweben J. E., Cohen J. B., Christian D. (2004). Psychiatric symptoms in methamphetamine users. *American Journal on Addictions*.

[45] Curran C., Byrappa N., McBride A. (2004). Stimulant psychosis: systematic review. *The British Journal of Psychiatry*.

[46] Farrell M., Boys A., Bebbington P. (2002). Psychosis and drug dependence: Results from a national survey of prisoners. *The British Journal of Psychiatry*.

[47] Graf K., Baer P. E., Comstock B. S. (1977). Mmpi changes in briefly hospitalized non-narcotic drug users. *The Journal of Nervous and Mental Disease*.

[48] Dalmau A., Bergman B., Brismar B. (1999). Psychotic disorders among inpatients with abuse of cannabis, amphetamine and opiates. Do dopaminergic stimulants facilitate psychiatric illness?. *European Psychiatry*.

[49] Walter M., Dammann G., Wiesbeck G. A., Klapp B. F. (2005). Psychosocial stress and alcohol consumption: Interrelations, consequences and interventions. *Fortschritte der Neurologie Psychiatrie*.

[50] Kranzler H. R., Mulgrew C. L., Modesto-Lowe V., Burleson J. A. (1999). Validity of the Obsessive Compulsive Drinking Scale (OCDS): Does craving predict drinking behavior?. *Alcoholism: Clinical and Experimental Research*.

[51] Helzer J. E., Pryzbeck T. R. (1988). The co-occurrence of alcoholism with other psychiatric disorders in the general population and its impact on treatment. *Journal of Studies on Alcohol*.

[52] Tran B. X., Nguyen L. H., Nong V. M., Nguyen C. T. (2016). Health status and health service utilization in remote and mountainous areas in Vietnam. *Health and Quality of Life Outcomes*.

[53] Nguyen L. H., Tran B. X., Nguyen H. L. T. (2017). Psychological Distress Among Methadone Maintenance Patients in Vietnamese Mountainous Areas. *AIDS and Behavior*.

[54] Vuong D. A., Van Ginneken E., Morris J., Ha S. T., Busse R. (2011). Mental health in Vietnam: Burden of disease and availability of services. *Asian Journal of Psychiatry*.

[55] Van Nguyen H., Nguyen H. L., Mai H. T. (2017). Stigmatization among methadone maintenance treatment patients in mountainous areas in northern Vietnam. *Harm Reduction Journal*.

[56] Des Jarlais D., Duong H. T., Pham Minh K. (2016). Integrated respondent-driven sampling and peer support for persons who inject drugs in Haiphong, Vietnam: a case study with implications for interventions. *AIDS Care Psychological and Socio-medical Aspects of AIDS/HIV*.

[57] Uthman O. A., Magidson J. F., Safren S. A., Nachega J. B. (2014). Depression and adherence to antiretroviral therapy in low-, middle- and high-income countries: a systematic review and meta-analysis. *Current HIV/AIDS Reports*.

[58] Ho R. C., Zhang M. W. B., Tsang T. Y. (2014). The association between internet addiction and psychiatric co-morbidity: A meta-analysis. *BMC Psychiatry*.

[59] Ho R. C. M., Ho E. C. L., Tan C. H., Mak A. (2009). Pulmonary hypertension in first episode infective endocarditis among intravenous buprenorphine users: Case report. *American Journal of Drug and Alcohol Abuse*.

[60] Ho R. C. M., Ho E. C. L., Mak A. (2009). Cutaneous complications among i.v. buprenorphine users. *The Journal of Dermatology*.

